# Remodelin delays non‐small cell lung cancer progression by inhibiting NAT10 via the EMT pathway

**DOI:** 10.1002/cam4.7283

**Published:** 2024-06-03

**Authors:** Quanwei Guo, Weijun Yu, Jianfeng Tan, Jianhua Zhang, Jin Chen, Shuan Rao, Xia Guo, Kaican Cai

**Affiliations:** ^1^ The First School of Clinical Medicine Southern Medical University Guangzhou China; ^2^ Department of Thoracic Surgery, Shenzhen Hospital Southern Medical University Shenzhen China; ^3^ Bao'an District Hospital for Chronic Diseases Prevention and Cure Shenzhen China; ^4^ Science and Education Department, Shenzhen Hospital Southern Medical University Shenzhen China; ^5^ Department of Thoracic Surgery, Nanfang Hospital Southern Medical University Guangzhou China; ^6^ Center for Clinical Research and Innovation, Shenzhen Hospital Southern Medical University Shenzhen China; ^7^ Shenzhen Key Laboratory of Viral Oncology Shenzhen China

**Keywords:** epithelial‐to‐mesenchymal transition, invasion and metastasis, lung cancer, mouse xenograft, N‐acetyltransferase 10, patients‐derived organoids, proliferation, Remodelin, tumorigenicity

## Abstract

**Background:**

Lung cancer remains the foremost reason of cancer‐related mortality, with invasion and metastasis profoundly influencing patient prognosis. N‐acetyltransferase 10 (NAT10) catalyzes the exclusive N (4)‐acetylcytidine (ac4C) modification in eukaryotic RNA. NAT10 dysregulation is linked to various diseases, yet its role in non‐small cell lung cancer (NSCLC) invasion and metastasis remains unclear. Our study delves into the clinical significance and functional aspects of NAT10 in NSCLC.

**Methods:**

We investigated NAT10's clinical relevance using The Cancer Genome Atlas (TCGA) and a group of 98 NSCLC patients. Employing WB, qRT‐PCR, and IHC analyses, we assessed NAT10 expression in NSCLC tissues, bronchial epithelial cells (BECs), NSCLC cell lines, and mouse xenografts. Further, knockdown and overexpression techniques (siRNA, shRNA, and plasmid) were employed to evaluate NAT10's effects. A series of assays were carried out, including CCK‐8, colony formation, wound healing, and transwell assays, to elucidate NAT10's role in proliferation, invasion, and metastasis. Additionally, we utilized lung cancer patient‐derived 3D organoids, mouse xenograft models, and Remodelin (NAT10 inhibitor) to corroborate these findings.

**Results:**

Our investigations revealed high NAT10 expression in NSCLC tissues, cell lines and mouse xenograft models. High NAT10 level correlated with advanced T stage, lymph node metastasis and poor overall survive. NAT10 knockdown curtailed proliferation, invasion, and migration, whereas NAT10 overexpression yielded contrary effects. Furthermore, diminished NAT10 levels correlated with increased E‐cadherin level whereas decreased N‐cadherin and vimentin expressions, while heightened NAT10 expression displayed contrasting results. Notably, Remodelin efficiently attenuated NSCLC proliferation, invasion, and migration by inhibiting NAT10 through the epithelial‐mesenchymal transition (EMT) pathway.

**Conclusions:**

Our data underscore NAT10 as a potential therapeutic target for NSCLC, presenting avenues for targeted intervention against lung cancer through NAT10 inhibition.

## 1 INTRODUCTION

Lung cancer represents a primary malignancy globally and stands as the foremost reason of cancer‐related mortality.^1^ Non‐small cell lung cancer (NSCLC) accounts for approximately 85% of all lung cancer cases.^2^, ^3^ Despite substantial therapeutic advancements encompassing surgery, chemotherapy, molecular targeting therapies, and immunotherapy, the persistence of invasion and metastasis remains a formidable challenge in NSCLC treatment.^4^, ^5^, ^6^ Hence, there is an imperative need for continued research into novel drugs and therapies aimed at extending clinical benefits to a wider patient cohort and improving outcomes in NSCLC.

NAT10, also known as human N‐acetyltransferase‐like protein or Kre33, is a member of the general control non‐derepressible 5 (GCN5) related N‐acetyltransferases.^7^ Initially identified as a regulator of telomerase activity, NAT10 is implicated in various cellular processes, such as the DNA damage response, cytokinesis, and modifications of histones and microtubules.^8^, ^9^, ^10^, ^11^, ^12^ NAT10 dysregulation has been observed in diseases such as Hutchinson‐Gilford progeria syndrome and some cancer types, including colorectal, hepatocellular carcinoma, pancreatic cancer and melanoma.^13^, ^14^, ^15^, ^16^, ^17^, ^18^ Notably, in human colorectal carcinomas, NAT10 was found to play a pivotal role in p53 activation by acetylating p53 and offsetting Mdm2 action.^9^ However, limited information exists regarding the role of NAT10 in invasion and metastasis in NSCLC. Previous research from our group aimed to demonstrate that downregulation of NAT10 expression might impede NSCLC tumorigenesis and progression.

Remodelin, an inhibitor of NAT10, has shown potential in mediating nuclear shape restoration in laminopathic cells through microtubule reorganization.^19^ Recent studies have suggested the possible clinical utility of Remodelin in augmenting the efficacy of doxorubicin‐based chemotherapy in breast cancer.^20^ Additionally, Remodelin has exhibited inhibitory effects on invasion and migration of hepatocellular carcinoma cells under hypoxic conditions.^15^ Furthermore, Remodelin could inhibit HIV‐1 replication without affecting cell viability and change mitochondrial lipid metabolism in cancer cells.^21^, ^22^ Nonetheless, the impact of Remodelin specifically in NSCLC remains unexplored.

## 2 MATERIALS AND METHODS

### 2.1 Patients and tissue samples

Ninety‐eight NSCLC patients between January 2004 and December 2009 contributed 180 NSCLC tissues and adjacent normal tissues to the tissue chip used in this study. Histological confirmation of NSCLC was conducted by three independent histopathologists. Fresh NSCLC tissues were obtained from two patients for organoid culture at Shenzhen Hospital, Southern Medical University, between August 2020 and September 2020.

### 2.2 NAT10 inhibitor and antibodies

Remodelin, a NAT10 inhibitor (S7641, Selleckchem, USA), was employed in this study. Monoclonal antibody NAT10 was procured from ABclonal Group (A7292, Boston, USA). Monoclonal antibodies against E‐Cadherin, N‐Cadherin, and Vimentin were purchased from CST Group.

### 2.3 Tissue processing and organoid development

Fresh NSCLC tumor tissues underwent initial dissection and cleansing with cold PBS containing antibiotics before being cut into 5 mm^3^ segments. Subsequently, the tumor segments were washed with Advanced DMEM/F12. The tissue fragments were enzymatically digested in a 10 mL medium comprising 2% fetal calf serum and 2 mg/mL collagenase while agitating on a shaker at 37°C for 1–2 h. The digested material was centrifuged at 400 × g for 4 min. Following centrifugation, the pellet was washed, resuspended, and centrifuged again at 400 × g for 3 min. The dissociated cells were suspended in Advanced DMEM/F12 mixed with growth factor‐reduced Matrigel and allowed to solidify at 37°C for 30 min. A 500 μL volume of complete human organoid medium (HOM) was layered onto the solidified cell suspension/Matrigel mixture. HOM was replenished every 2–3 days throughout the 1‐week organoid development period. Passage of organoids was conducted weekly upon reaching sizes ranging from 200 to 500 μm. Organoids were dissociated and passaged using TrypLE Express (Gibco). For long‐term preservation, living organoids (2 × 10^6^ cells/tube) were banked in Recovery Cell Culture Freezing Medium (Gibco) at −80°C.

### 2.4 Cell lines, culturing, transfection, and proliferation assay

Human bronchial epithelial cells, A549, and NCI‐H1975 lung cancer cell lines were acquired from the Chinese academy of sciences cell bank and maintained in DMEM at 37°C in a 5% CO_2_ atmosphere. For loss‐of‐function studies, cells were transfected with NAT10siRNA or NAT10shRNA (GenePharma, Shanghai, China). Gain‐of‐function experiments involved transfection of A549 and NCI‐H1975 cells with OE‐NAT10 vector plasmids (Youbio Biological Technology Co., Ltd., Beijing, China). Lipofectamine 2000 was utilized for transfection following manufacturer's instructions.

As for the lentiviral transfection, the cells were plated into the 24‐well plate (1 × 10^5^/well) overnight, then 2 mL of fresh medium containing appropriate volum of virus suspension was added for 48–72 h. Lastly, 1 μg/mL of puromycin was added into medium and dosing can be started 3–4 days after transfection.

For the proliferation assay, A549 and NCI‐H1975 cells were prepared a cell suspension of 3 × 10^4^ cells/mL. Subsequently, 100 μL of each cell suspension was seeded in triplicate into 96‐well culture plates and incubated overnight at 37°C in a 5% CO_2_ incubator. Cell Counting Kit‐8 reagent mixed with serum‐free RPMI‐1640 medium was added at a ratio of 1:10, followed by 1‐h incubation at 37°C. Absorbance at 450 nm was measured. Statistical analysis was performed after conducting three independent assays.

### 2.5 Wound healing assay

5 × 10^4^ A549 and NCI‐H1975 cells were seeded in a 24‐well plate and cultured for 2 days. A 1 mL wound pipette tip was used to cut the middle of each well. Subsequently, floating cells were eliminated by three washes with medium, and fresh medium supplemented with 1% FBS was added. Images were captured at 0, 12, 24 and 48 h. Statistical analysis was performed following three independent assays.

### 2.6 Transwell assay

Matrigel‐coated transwell chambers were employed. 5 × 10^4^ cells were seeded into the upper chamber. Cells on the upper membrane surface were removed after 2 days incubation, whereas cells that migrated to the lower side were fixed and stained using crystal violet. The assay was independently conducted six times, and statistical analysis was performed accordingly.

### 2.7 Clone formation assay

A549 and NCI‐H1975 cells were seeded into 96‐well plates at a density of 1 × 10^2^ cells/well. After a 7‐day incubation period, visible colonies were fixed with methanol and stained with crystal violet solution. Colony counts were performed using an optical microscope (×100). This assay was repeated three times for validation purposes.

### 2.8 Real‐time reverse transcription polymerase chain reaction (qRT‐PCR)

Total RNA was extracted from lung cancer cell lines using TRIzol Reagent. Reverse transcription of RNA to cDNA was conducted using a reverse transcription kit. NAT10 expression was assessed using β‐actin as the reference gene. Each experiment was conducted in triplicate.

### 2.9 Western blotting (WB)

Cells subjected to respective treatments were lysed in a lysis buffer. Protein concentrations were detected using a BCA protein assay. Approximately 20 μg of protein was resolved via SDS‐PAGE and transferred onto a PVDF membrane. The membrane was blocked with 5% nonfat milk, incubated with primary antibodies against NAT10, E‐cadherin, N‐cadherin, vimentin, and GAPDH. Subsequently, the blots were probed with an HRP‐conjugated secondary antibody, and the chemiluminescent signal was quantified using Image J.

### 2.10 Immunochemistry (IHC)

Paraffin‐embedded sections of NSCLC tissue specimens were initially subjected to dewaxing and dehydration using a gradient of alcohol. The sections underwent a 20‐min incubation with 3% H_2_O_2_‐methanol and were subsequently rinsed with 0.1 M PBS for 3 min. Antigen retrieval was conducted using a solution in a water bath. Following this, a normal goat serum blocking solution was applied to the sections and allowed to incubate at room temperature for 20 min. Excess solution was removed. Overnight incubation was conducted by applying primary rabbit anti‐human antibodies against NAT10 (A7292, ABclonal, 1:1000), E‐cadherin (CST3195, 1:1000), N‐cadherin (CST13116, 1:1000), and vimentin (CST#5741P, 1:1000) at 4°C. Subsequent to this, incubation with horseradish peroxidase (HRP)‐conjugated secondary antibody was carried out at 37°C for 20 min. Color development was achieved using diaminobenzidine (DAB), followed by counterstaining with hematoxylin for 1 min. The sections were reblued using 1% ammonia, followed by dehydration using a graded alcohol series, clearing in xylene, and mounting using neutral resin. The mounted slides were observed and imaged under a microscope.

### 2.11 Animal experiments

Ethical approval was obtained from the Shenzhen Hospital of Southern Medical University Institutional Animal Use and Care Committee. Five‐week‐old BALB/c nude mice were subcutaneously injected with 5 × 10^6^ A549 cells in 0.1 mL suspension into the right armpit region. All mice were randomly divided into four groups (*n* = 6). Tumor size was measured every 4 days using calipers. After 29 days post‐injection, the mice were euthanized, and subcutaneous tumors were excised, measured for size, and weighed.

For gavage feeding, a 0.2 mL solution containing 20 mg Remodelin was administered daily to each 20 g mouse (equivalent to 100 mg/kg body weight) from Day 22 to Day 29 of the experiment.

### 2.12 Statistical analysis

Statistical analyses were performed using SPSS software package and GraphPad Prism 9.0. Differences between two groups were assessed using Student's *t*‐test, while multiple group comparisons were conducted using one‐way analysis of variance (ANOVA). The significance level was set at *p* < 0.05.

### 2.13 Ethical statement

This study received approval from the Ethics Committee of Shenzhen Hospital of Southern Medical University. All participating patients had signed informed consent before enrollment, adhering to the guidelines stipulated in the Helsinki Declaration.

## 3 RESULTS

### 3.1 NAT10 overexpression in NSCLC tissues and cell lines

**FIGURE 1:**
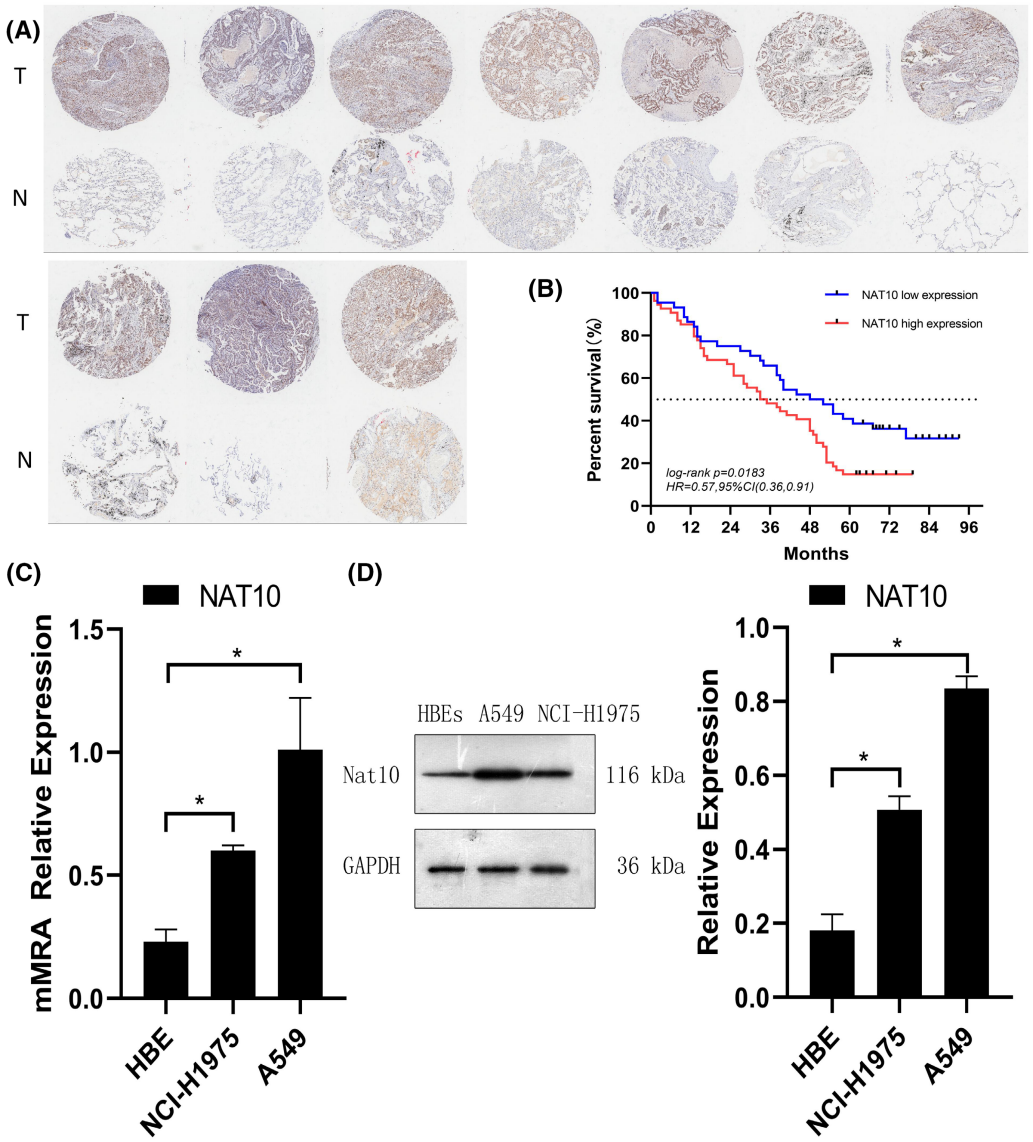
High NAT10 Expression in NSCLC. (A) Representative immunohistochemical images depicting NAT10 expression in NSCLC tissues (10 cases) and normal lung tissues (10 cases) from the Tissue Microarray (TMA). (B) Correlation between high NAT10 expression and Overall Survival (OS) in NSCLC patients. (C) Quantitative analysis of NAT10 expression levels in human bronchial epithelial cells (BECs) and NSCLC cancer cell lines (A549 and NCI‐H1975) conducted via qRT‐PCR. β‐Actin was used as the reference gene for normalization. (D) Western blot analysis illustrating NAT10 expression levels in BECs and NSCLC cell lines. GAPDH was utilized as a loading control in the western blotting assays. Values are depicted as the mean ± SD of three independent experiments.

The investigation commenced by evaluating NAT10 expression using IHC on an NSCLC tissue microarray (TMA) comprising 180 samples from 98 NSCLC patients, alongside adjacent normal tissues (Table S1). The findings unveiled predominant nuclear localization of NAT10, with significantly higher expression levels observed in NSCLC tissues compared to adjacent normal tissues (Figure 1A). Moreover, Kaplan–Meier survival analysis exhibited a noteworthy correlation between elevated NAT10 expression and overall survival (*p* = 0.0183) (Figure 1B). Additionally, high NAT10 expression correlated with advanced T stage and lymph node metastasis (Table S2).

Subsequently, the mRNA and protein expression levels of NAT10 were assessed in BECs and NSCLC cell lines (A549 and NCI‐H1975) using qRT‐PCR and WB. Both NSCLC cell lines demonstrated markedly higher NAT10 mRNA and protein expression compared to BECs, consistent with the observations in the tissue microarray (Figure 1C,D). These comprehensive findings collectively underscore the prognostic significance of NAT10 and its potential oncogenic implications in NSCLC.

### 3.2 Down‐ and up‐regulation of NAT10 in NSCLC cell lines

To delineate the biological function of NAT10 in NSCLC tumorigenesis, NAT10‐knockdown cells were generated using siRNAs, and NAT10‐overexpression cells were established via overexpression plasmids in A549 and NCI‐H1975 cell lines. The efficacy of siRNA‐mediated knockdown and plasmid‐induced overexpression of NAT10 was validated through qRT‐PCR and WB.

**FIGURE 2:**
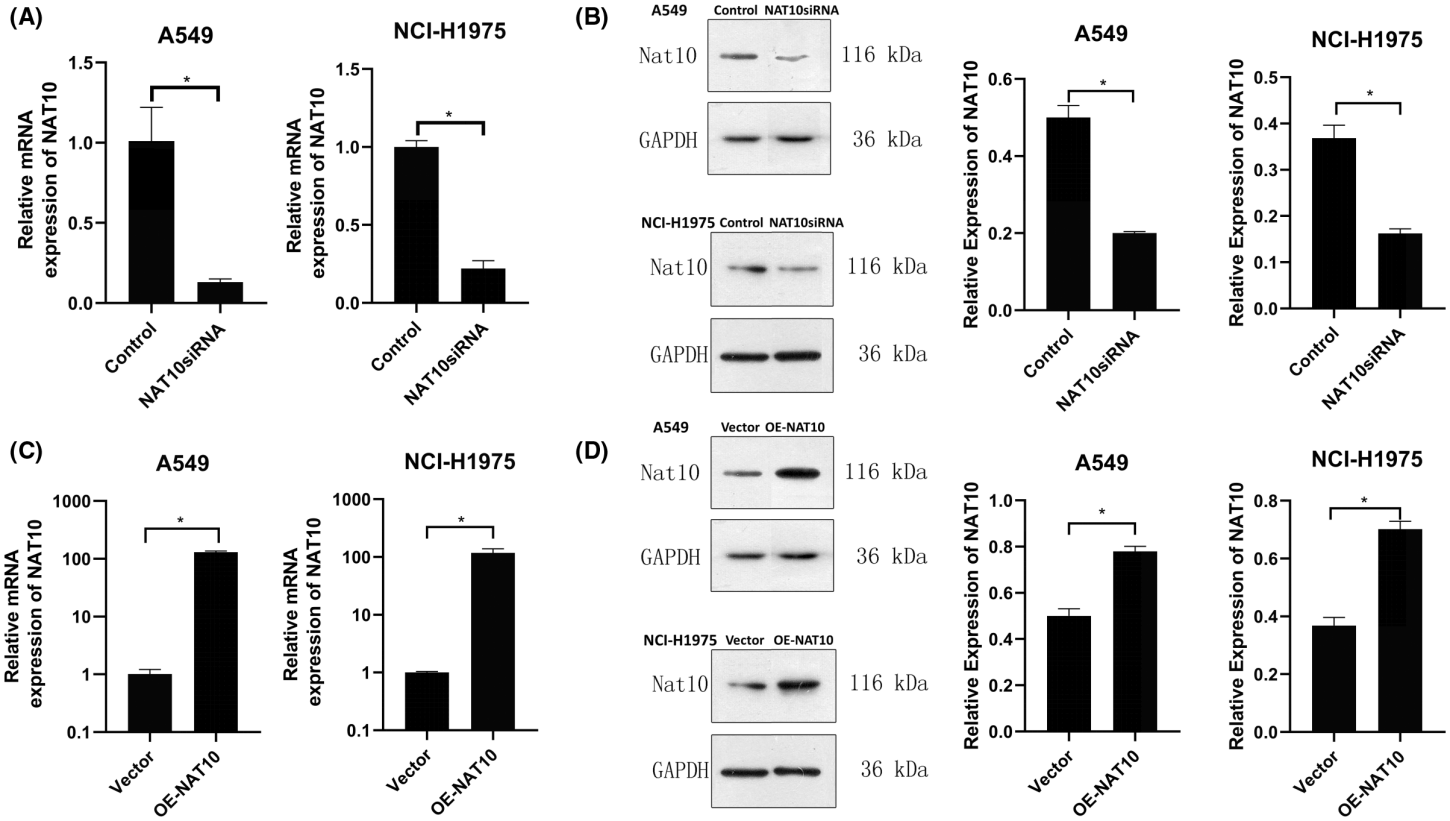
Modulation of NAT10 Expression in Lung Cancer Cell Lines. (A) Quantitative reverse transcription‐polymerase chain reaction (qRT‐PCR) analysis illustrating NAT10 expression levels in NSCLC cell lines following transfection with NAT10siRNA *p < 0.05 versus Control. (B) Western blotting showing NAT10 expression levels in NSCLC cell lines post‐transfection with NAT10siRNA. *p < 0.05 versus Control. (C) qRT‐PCR analysis demonstrating NAT10 expression levels in NSCLC cell lines transfected with OE‐NAT10. *p < 0.05 versus Vector. (D) Western blotting depicting NAT10 expression levels in NSCLC cell lines after transfection with OE‐NAT10. *p < 0.05 versus Vector. β‐actin was utilized as the reference gene for qRT‐PCR, while GAPDH served as the loading control in Western blotting assays. The depicted values represent the mean ± standard deviation (SD) obtained from three independent experiments.

NAT10siRNA successfully reduced both the mRNA and protein expression of NAT10 compared to the control group, as depicted in Figure 2A,B. Conversely, transfection with the OE‐NAT10 plasmid significantly increased the mRNA and protein expression levels of NAT10 in both lung cancer cells compared to the vector control, as shown in Figure 2C,D.

### 3.3 NAT10 promotes proliferation, tumorigenicity, invasion, and migration of NSCLC cell lines

**FIGURE 3:**
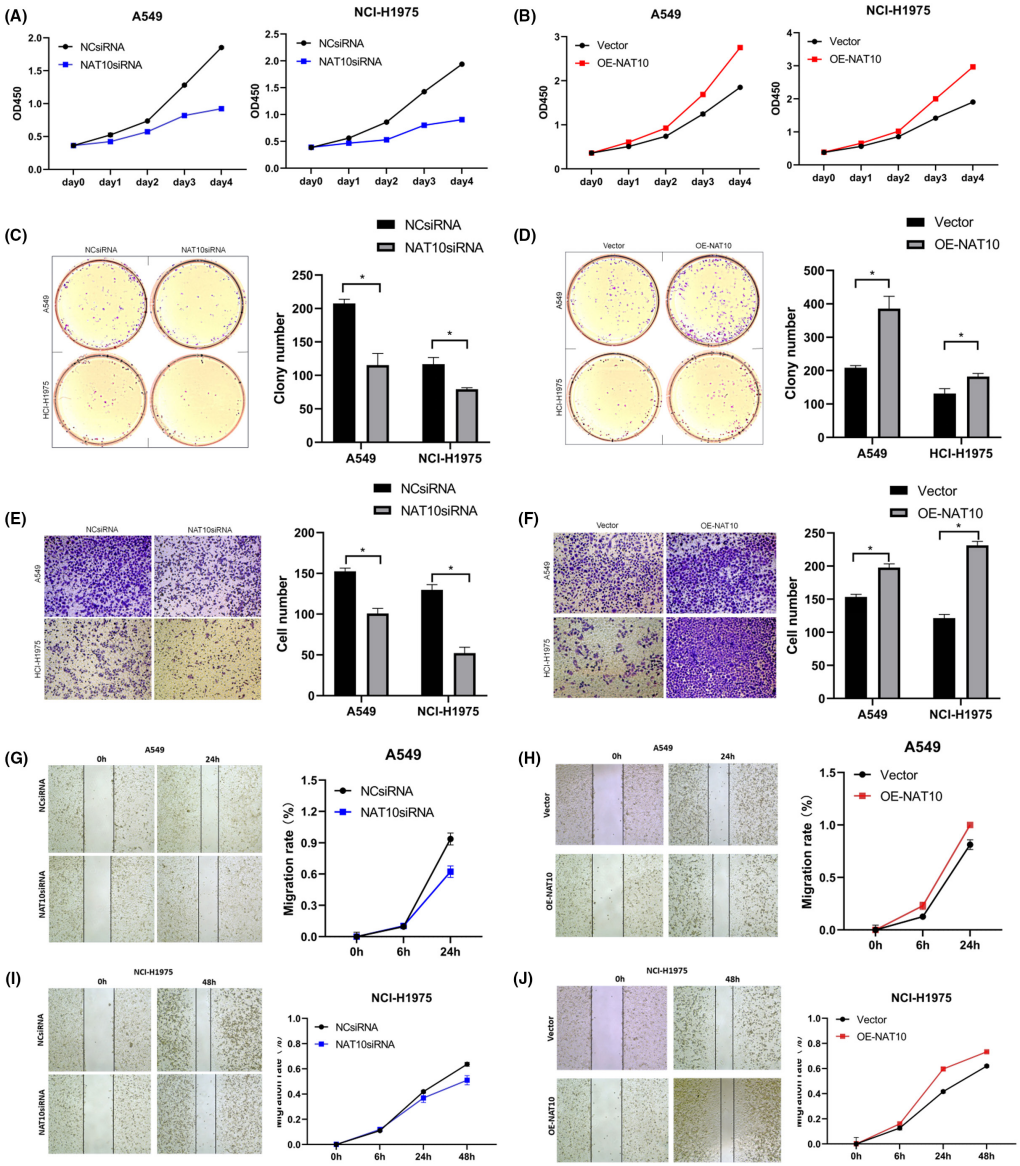
NAT10 Promotes Proliferation, Tumorigenicity, Invasion, and Migration in NSCLC Cell Lines. (A and B) Quantification of Cell Counting Kit‐8 (CCK8) assays depicting the viability of NSCLC cell lines post‐transfection with NCsiRNA, NAT10siRNA, Vector, and OE‐NAT10. *p < 0.05. (C and D) Images and quantification of clone formation assays representing the clonogenic potential of NSCLC cell lines following transfection with NCsiRNA, NAT10siRNA, Vector, and OE‐NAT10. *p < 0.05. (E and F) Images and quantification of transwell assays demonstrating the invasive capacity of NSCLC cell lines subsequent to transfection with NCsiRNA, NAT10siRNA, Vector, and OE‐NAT10. *p < 0.05. (G–J) Images and quantification of wound healing assays illustrating the migratory ability of NSCLC cell lines post‐transfection with NCsiRNA, NAT10siRNA, Vector, and OE‐NAT10. *p < 0.05. GAPDH served as a loading control. The depicted values denote the mean ± standard deviation (SD) obtained from three independent experiments.

To elucidate NAT10's impact on NSCLC cell viability, cell counting kit‐8 (CCK8) assays were conducted. Upon downregulation of NAT10 expression in A549 and NCI‐H1975 cells through siRNA transfection, the viability of NAT10‐silenced cells was assessed at 0, 24, 48, 72, and 96 h. The results revealed a notable inhibition in the viability of A549 and NCI‐H1975 cell lines following NAT10siRNA transfection (Figure 3A). Conversely, the upregulation of NAT10 in the OE‐NAT10 group significantly augmented the cell viability of A549 and NCI‐H1975 cell lines (Figure 3B). The results strongly suggest that NAT10 plays a key role in promoting the viability of NSCLC cells, as observed through the differential modulation of NAT10 expression levels.

Proliferation and tumorigenicity were assessed using the colony formation assay. Decreased expression of NAT10 resulted in a notable suppression of proliferation and tumorigenicity in A549 and NCI‐H1975 cells compared to the control group (Figure 3C). Conversely, increased expression of NAT10 promoted proliferation and tumorigenicity in both NSCLC cell lines in contrast to the vector group (Figure 3D), corroborating findings from the CCK‐8 assay. These outcomes strongly suggest that NAT10 amplifies the proliferation and tumorigenic potential of NSCLC cell lines.

Investigating into the invasion and metastasis capabilities of NAT10 in NSCLC, transwell invasion and wound‐healing migration assays were performed. In the transwell invasion assay, fewer NSCLC cells transfected with NAT10siRNA traversed the membranes compared to the control group (Figure 3E). Conversely, NSCLC cells overexpressing OE‐NAT10 exhibited increased migration through the transwell membranes in comparison to the vector group (Figure 3F). Likewise, the wound‐healing migration assay depicted a wider wound distance in NSCLC cells transfected with NAT10siRNA, while the OE‐NAT10 group displayed a narrower wound distance than the NSCLC cells transfected with the vector (Figure 3G–J). These collective findings unequivocally demonstrate that NAT10 acts a crucial role in promoting invasion and migration in NSCLC cells.

### 3.4 NAT10 promotes NSCLC cell lines development via the EMT pathway

The EMT pathway is recognized as a crucial oncogenic mechanism involved in tumor initiation and progression. E‐cadherin, N‐cadherin, and Vimentin are fundamental signaling molecules in the EMT cascade. Thus, our hypothesis was that NAT10 might facilitate NSCLC cell proliferation, migration, invasion, and tumorigenicity through the EMT pathway in vitro. In our study, the expression of NAT10, E‐cadherin, N‐cadherin, and Vimentin were assessed in NCsiRNA, NAT10siRNA, vector, and OE‐NAT10 groups using qRT‐PCR and WB.

**FIGURE 4:**
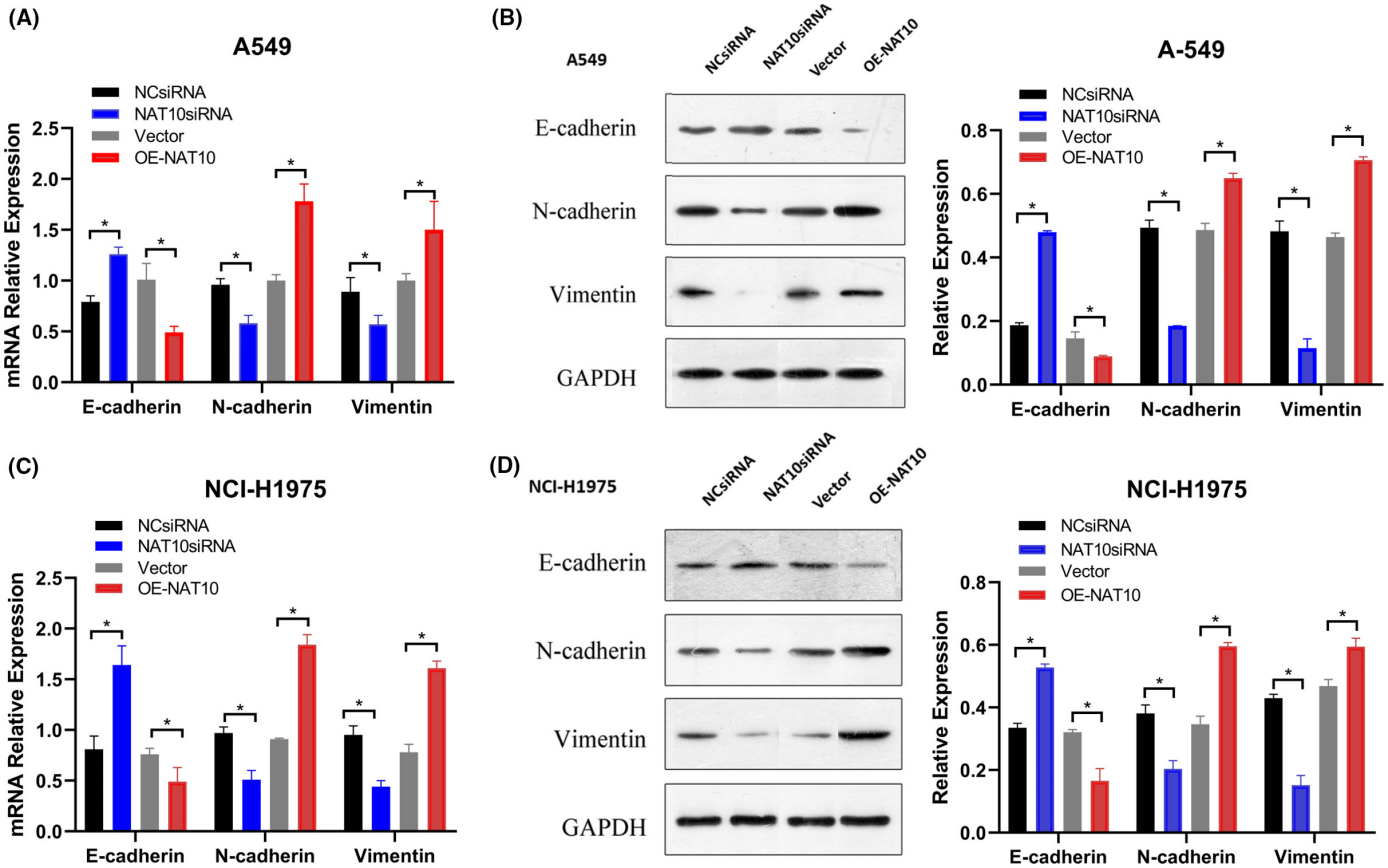
NAT10's Association with the EMT Phenotype in Human NSCLC Cell Lines. (A, C) Quantification of E‐ cadherin, N‐cadherin, and Vimentin expression in NSCLC cell lines assessed via qRT‐PCR analysis. *p < 0.05, indicating statistically significant differences. (B, D). Western blotting results displaying the expression levels of E‐cadherin, N‐cadherin, and Vimentin following NAT10 knockdown with NAT10‐siRNA and overexpression of NAT10 using OE‐NAT10. β‐Actin served as a reference gene in the qRT‐PCR analysis, while GAPDH was used as a loading control in the Western blotting. Values depicted are the mean ± SD obtained from three independent experiments.

As depicted in Figure 4A–D, both mRNA and protein levels of E‐cadherin noticeably increased in the NAT10siRNA group compared to the NCsiRNA group. Conversely, they notably decreased in the OE‐NAT10 groups contrasted with the vector group. Concerning the mesenchymal markers, the mRNA and protein levels of N‐cadherin and Vimentin significantly decreased in the NAT10siRNA group in comparison to the NCsiRNA group. However, they markedly increased in the OE‐NAT10 groups compared to the control group. These results collectively suggest that NAT10 likely regulates the proliferation, metastasis, and invasion of lung cancer cells through the modulation of EMT pathway activation.

### 3.5 Remodelin suppresses NSCLC progression by targeting NAT10 and regulating EMT

**FIGURE 5:**
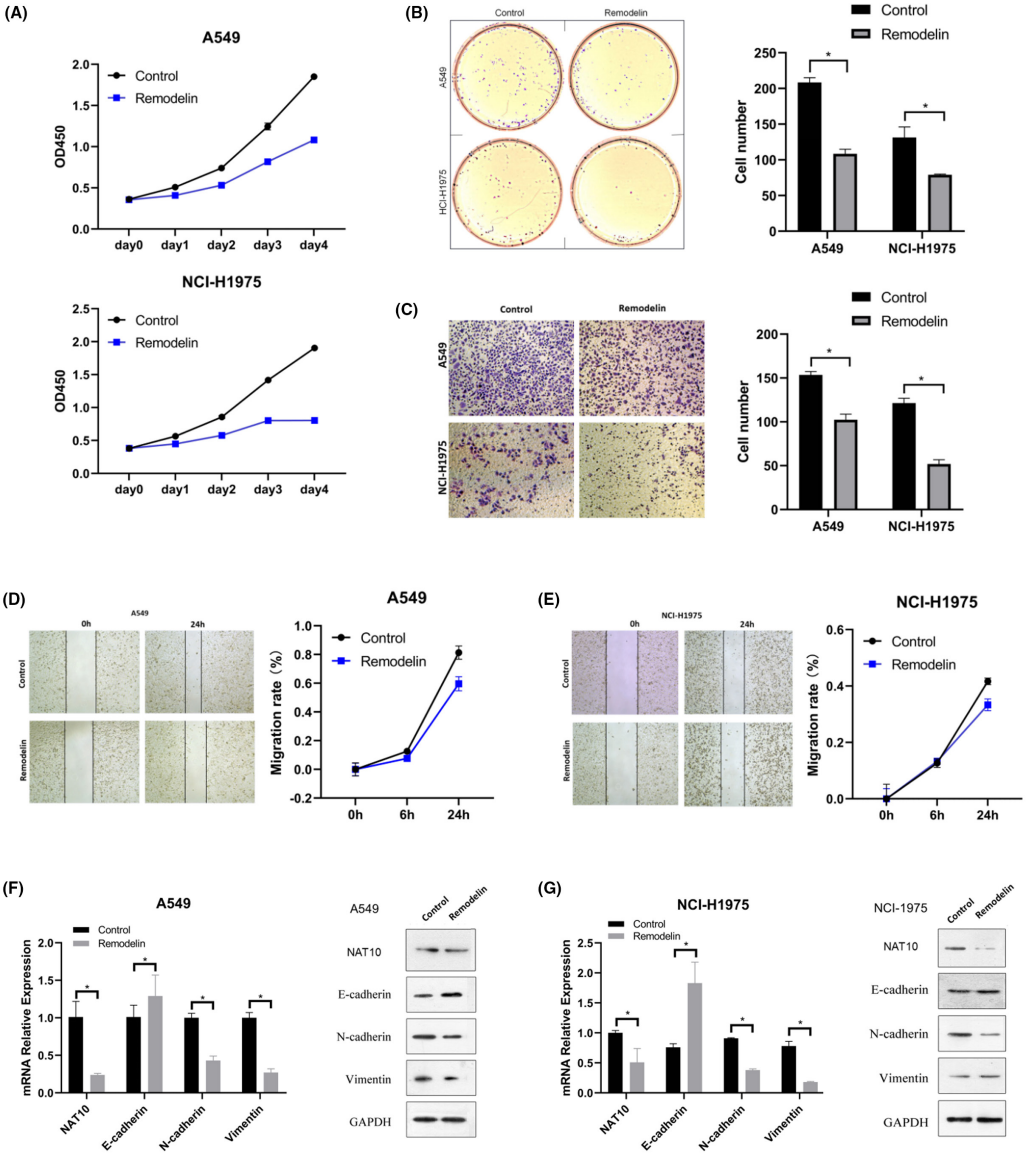
Remodelin's Impact on NSCLC Progression and EMT Phenotype (A) Quantification of CCK8 assays showing the effect of Remodelin treatment on NSCLC cell lines' proliferation. Statistical significance denoted by *p <0.05. (B) Images and quantification from clone formation assays depicting the influence of Remodelin treatment on NSCLC cell lines' clone formation ability. Statistical significance denoted by *p < 0.05. (C) Images and quantification demonstrating the impact of Remodelin treatment on transwell assays in NSCLC cell lines. Statistical significance denoted by *p < 0.05. (D and E) Images and quantification displaying the results of wound healing assays indicating the effect of Remodelin treatment on the migration capability of NSCLC cell lines. Statistical significance denoted by *p < 0.05. (F and G) Evaluation of E‐cadherin, N‐cadherin, and vimentin expression levels in NSCLC cell lines through qRT‐PCR and western blotting. β‐Actin served as the reference gene for qRT‐PCR, while GAPDH served as the loading control for western blotting. Values represented as mean ± SD from three independent experiments. Statistical analysis conducted using the Student's *t*‐test.

The impact of Remodelin on NSCLC was investigated through its effects on proliferation, invasion, migration, and tumorigenicity of NSCLC cell lines and patient‐derived lung cancer organoids. To ascertain Remodelin's inhibitory effect on NAT10 in NSCLC, various assays were conducted, including the CCK8, transwells, wound healing, and clone formation assays. As presented in Figure 5A–E, Remodelin treatment resulted in an obvious inhibition of proliferative capacity, invasion, migration ability, and tumorigenicity of NSCLC cell lines when compared to the control group. Further examination, illustrated in Figure 5F,G, revealed Remodelin's ability to down‐regulate both mRNA and protein expression levels of NAT10. Notably, NAT10 exhibited a regulatory role in the expression of EMT markers. It was observed that NAT10 upregulated expression levels of E‐cadherin while concurrently downregulating expression levels of N‐cadherin and Vimentin. These findings delineate the mechanism underlying Remodelin's effectiveness in suppressing NSCLC progression. Remodelin exhibited a dual impact by inhibiting NAT10 and modulating EMT markers, highlighting its potential as a promising therapeutic agent for managing NSCLC.

**FIGURE 6:**
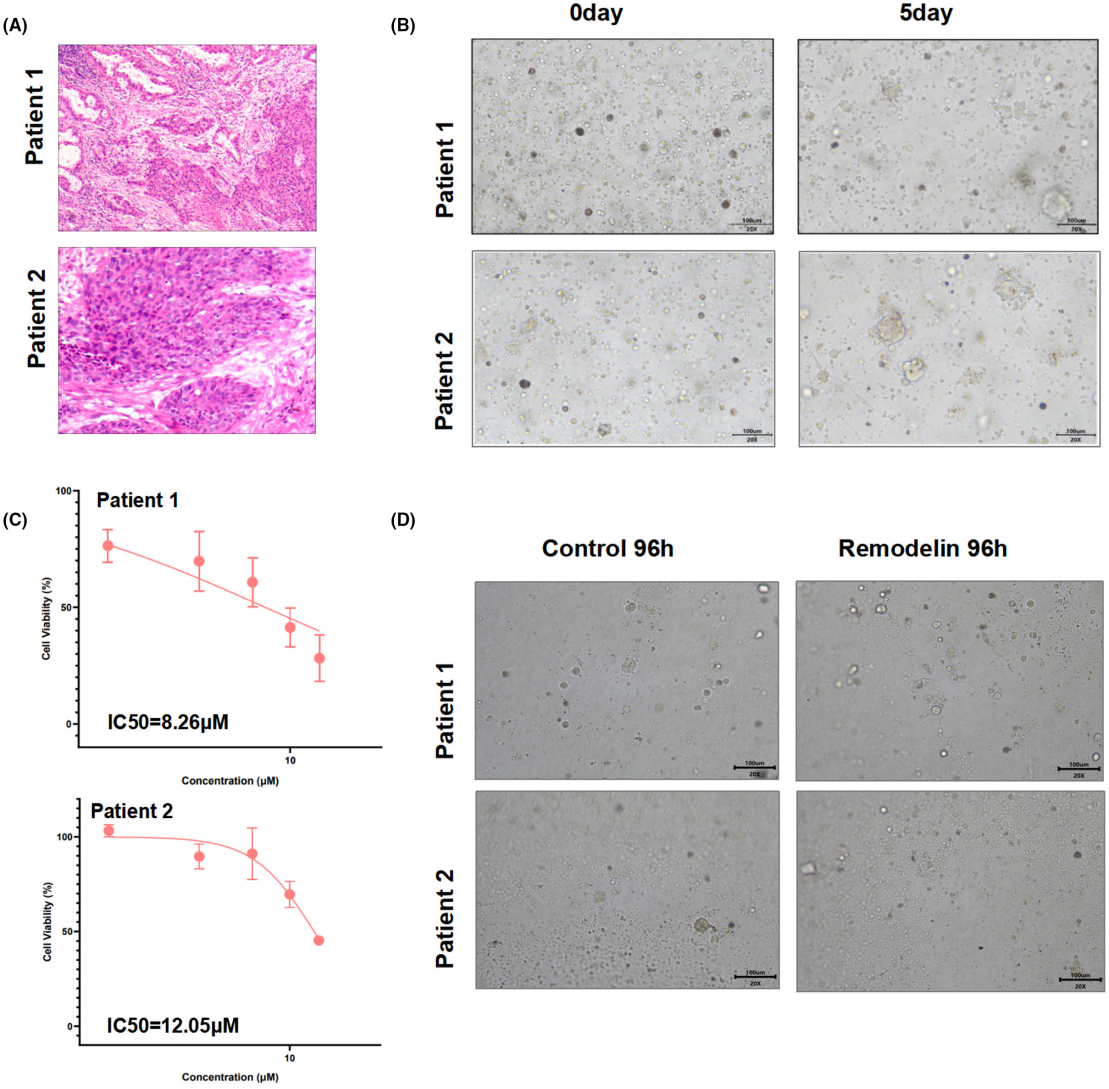
Remodelin's Effect on NSCLC Patient‐Derived Organoids. (A) Hematoxylin and eosin (HE) stained images depicting representative sections from two NSCLC patients. (B) Successful cultivation and growth of organoids derived from two NSCLC patients, establishing a platform for further experimentation. (C) Evaluation of proliferation and tumorigenesis inhibition efficiency of Remodelin in two distinct NSCLC patient‐derived organoids treated with varying concentrations. (D) Representative images displaying the impact of Remodelin treatment on the growth of two NSCLC patient‐derived organoids after a 96‐h period, comparing treated versus untreated conditions.

Furthermore, we obtained two fresh lung cancer specimens to develop organoids, one of which was confirmed by pathology as adenocarcinoma and the other as squamous cell carcinoma (Figure 6A). We tested the function of Remodelin in NSCLC patient‐derived organoids, and the results consisted of cell assays. Notably, Figure 6B confirms the successful cultivation of patient‐derived organoids, establishing a platform for further experimentation. Figure 6C,D illustrate that Remodelin treatment resulted in a notable attenuation of proliferation and tumorigenesis in two distinct lung cancer patient‐derived organoids. Remarkably, these outcomes paralleled the efficiency observed with NAT10siRNA treatment. Collectively, these findings underscore Remodelin's potential to mitigate proliferation, invasion, migration, and tumorigenicity in both NSCLC cell lines and lung cancer patient‐derived organoids. The observed effects are presumed to occur through the inhibition of NAT10‐mediated EMT.

### 3.6 Remodelin attenuates proliferation, invasion and metastasis abilities of NSCLC by inhibiting NAT10 through the EMT pathway in vivo

**FIGURE 7:**
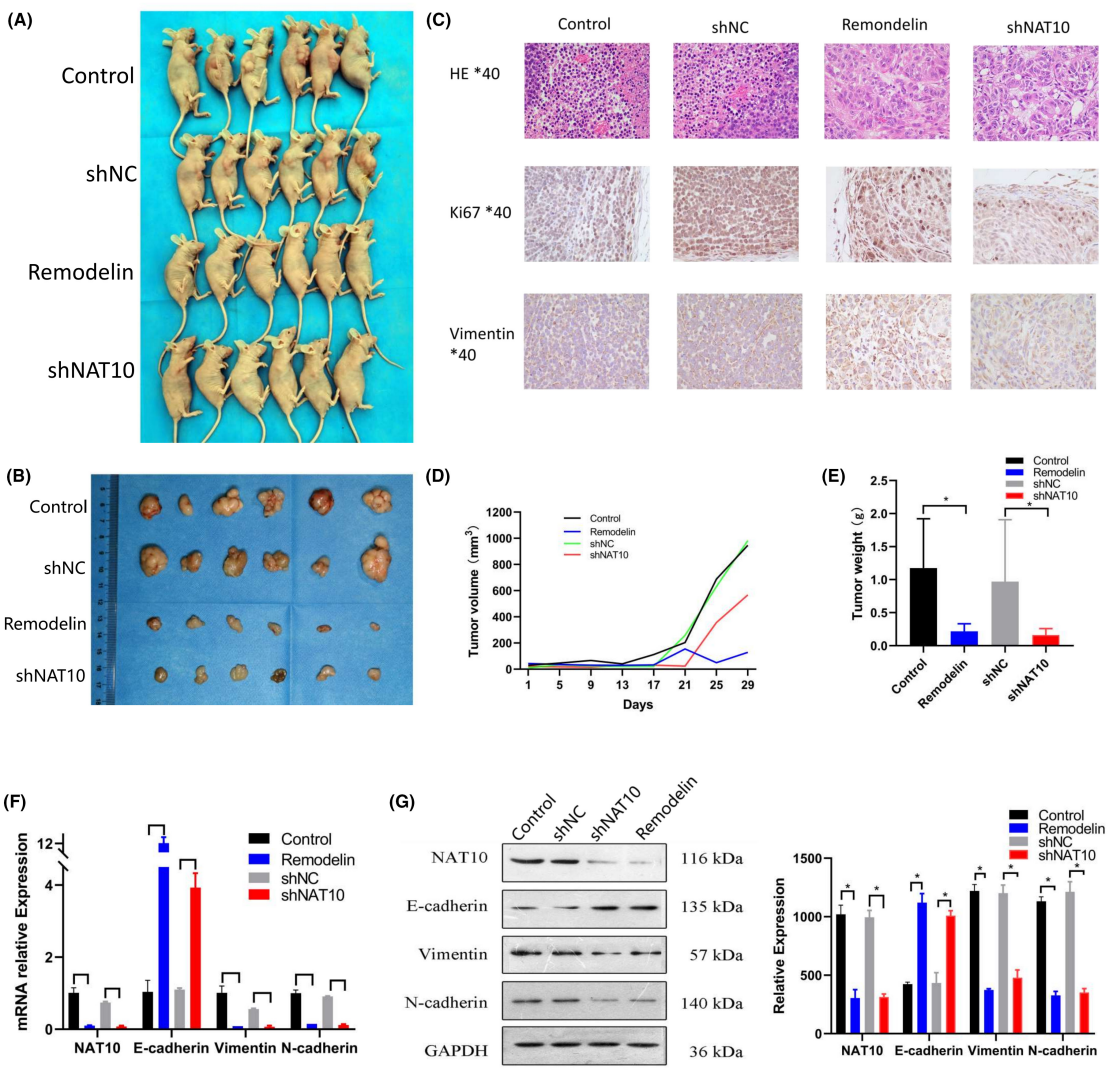
Remodelin's Effect on NSCLC Inhibition via NAT10 in Vivo. (A) Images displaying subcutaneous xenograft tumors (*n* = 6 for each group) derived from A549 cells under various treatment conditions. (B) Images of excised tumors from six BALB/c nude mice at 29 days after various treatment conditions. (C) Hematoxylin and eosin (HE) staining alongside Immunohistochemistry (IHC) of xenograft tumors, depicting the expression levels of Ki67 and Vimentin. (D, E) Evaluation of tumor volumes measured every 4 days and analysis of tumor weights in different experimental groups. (F, G) Examination of NAT10, E‐cadherin, N‐cadherin, and vimentin expression in xenograft tumors using both qRT‐PCR and western blotting techniques. β‐Actin served as the reference gene for qRT‐PCR, while GAPDH acted as the loading control in western blotting. Values represented as mean ± SD from three independent experiments. Statistical analysis conducted using Student's *t*‐test.

To investigate Remodelin's inhibitory effect on NAT10 in vivo, xenograft tumor models using A549 cells were established in nude mice. The experimental groups included A549 cells untreated, treated with Remodelin, stably transfected with shNC (non‐targeting control), and shNAT10. Analysis of tumor weight and volume trends demonstrated that NAT10 knockdown significantly inhibited tumor growth, with Remodelin exhibiting a similar effect comparable to shNAT10 (Figure 7A,B,D). IHC analysis revealed a notable reduction in Ki67 and Vimentin expression in the shNAT10 group contrary with the control, mirrored by similar outcomes in the Remodelin‐treated group (Figure 7C).

Moreover, qPCR and WB exhibited marked downregulation of N‐cadherin and Vimentin expression levels in both the shNAT10 and Remodelin‐treated groups (Figure 7E,F). These results from the animal models highlight the inhibitory effect of downregulated NAT10 expression on A549 cell tumorigenesis in vivo. Furthermore, Remodelin showcased its ability to attenuate proliferation, invasion, and metastasis abilities of NSCLC by inhibiting NAT10 through the EMT pathway, corroborating and extending the findings observed in vitro. The consistency between in vitro and in vivo observations suggests the potential therapeutic significance of Remodelin in managing NSCLC progression via NAT10 inhibition and modulation of the EMT pathway.

## 4 DISCUSSION

Lung cancer remains the foremost reason of cancer‐related mortality globally. Despite significant advancements in treatment modalities such as surgical procedures, chemotherapy regimens, radiation, targeted therapies, and immunotherapy, the 5‐year survival rates for lung cancer patients fluctuate between 4% and 17%, contingent upon stages and geographical disparities, especially among those with metastatic conditions.^4^, ^23^, ^24^, ^25^, ^26^ Notably, individuals diagnosed with Stage IV lung cancer exhibit a mere 5.8% 5‐year survival rate.^27^ Consequently, continued exploration of novel pharmaceuticals and therapies is imperative to extend clinical benefits to a wider NSCLC patient and ameliorate outcomes.

NAT10, a nucleolar protein comprising 1025 amino acids, encompasses an acetyltransferase domain and a lysine‐rich C‐terminus. The acetyltransferase domain facilitates the catalysis of N (4)‐acetylcytidine (ac4C) modification on mRNAs, 18S rRNA, and tRNAs.^28^, ^29^, ^30^ The presence of ac4C has been reported in the development, progression, and prognosis of various human diseases.^31^, ^32^, ^33^, ^34^ The mRNA ac4C modification has shown to enhance mRNA stability, translation, and translation efficiency.^30^ ac4C stands as the solitary acetylation event identified in eukaryotic RNA, and NAT10 is the singular human enzyme exhibiting both acetyltransferase and RNA binding activities.^28^ Despite the important role of NAT10 dysregulation in diverse diseases, limited information exists regarding its essential role in NSCLC and the impact of Remodelin.

Our research delineated the pivotal role of NAT10 in the tumorigenesis and progression of NSCLC. Recent scholarly reports underscored NAT10 as a crucial regulator in certain tumors. Zhang et al. observed upregulated NAT10 levels and its correlation with increased invasiveness and poorer clinical outcomes in colorectal cancer.^14^ Similarly, Ma et al. reported the promotion of metastasis in hepatocellular carcinoma via NAT10 upregulation and the induction of EMT.^15^ Our current investigation evidenced heightened NAT10 expression in NSCLC compared to adjacent normal tissues, correlating with T stage, lymph node metastasis, and dismal prognosis. Notably, NAT10 promoted proliferation, invasion, and metastasis in NSCLC cell lines, patient‐derived organoids, and xenograft tumor models. Furthermore, our findings corroborated Ma et al.'s observations, indicating NAT10's influence on E‐cadherin, N‐cadherin, and Vimentin expression levels.^15^

Remodelin was identified as a specific inhibitor targeting NAT10 by Larrieu in 2014.^17^ Notably, Remodelin exhibited efficacy in ameliorating health span, enhancing fitness, and delaying phenotypic manifestations of Hutchinson‐Gilford Progeria Syndrome (HGPS) in an HGPS mouse model by rebalancing NAT10.^35^ In this study, the role of Remodelin was investigated in NSCLC cell lines, patient‐derived organoids, and xenograft tumor models. Our comprehensive assays revealed Remodelin's efficacy in inhibiting NAT10, thereby reducing NSCLC proliferation, invasion, and metastatic capabilities. Additionally, molecular assays involving qRT‐PCR and WB suggested Remodelin's potential in suppressing the EMT by modulating its representative markers like E‐cadherin, N‐cadherin, and Vimentin.

However, our research only focused on highlighting the positive aspects of the study's findings, and did not address the potential challenges and barriers associated with translating preclinical findings into clinical applications, such as drug toxicity, off‐target effects, and drug resistance. More research is needed to determine whether NAT10 is a biomarker that can be used to target NSCLC treatment.

## 5 CONCLUSION

In summary, our study elucidated NAT10's oncogenic role in NSCLC progression. Furthermore, Remodelin demonstrated its potential to mitigate NSCLC proliferation, invasion, and metastasis by targeting NAT10 via the EMT pathway. Consequently, the targeting of NAT10 emerges as a promising therapeutic strategy for managing NSCLC.

## AUTHOR CONTRIBUTIONS

**Quanwei Guo:** Conceptualization (lead); funding acquisition (lead); project administration (equal); writing – original draft (lead). **Weijun Yu:** Formal analysis (lead); methodology (lead); software (lead); writing – original draft (equal). **Jianfeng Tan:** Data curation (lead); investigation (lead); validation (equal). **Jianhua Zhang:** Data curation (equal); funding acquisition (equal); resources (lead). **Jin Chen:** Formal analysis (equal); investigation (equal); software (equal). **Shuan Rao:** Supervision (equal); visualization (equal); writing – review and editing (lead). **Xia Guo:** Conceptualization (equal); methodology (equal); validation (lead); visualization (lead). **Kaican Cai:** Project administration (lead); resources (equal); supervision (lead); writing – review and editing (equal).

## FUNDING INFORMATION

This work was supported by National Nature Science Foundation of China (82103512), Research Fund of Shenzhen Hospital of Southern Medical University (PY2021ZY01), Young Innovative Talents Project of General Universities in Guangdong Province (2021KQNCX009).

## CONFLICT OF INTEREST STATEMENT

The authors have no conflicts of interest to declare.

## ETHICAL APPROVAL STATEMENT

All of the animal experiments in this work have received animal ethical permission from the Experimental Animal Center of Shenzhen hospital, Southern Medical University, Shenzhen, China.

## Supporting information


Table S1:

Table S2:

Table S3:

## Data Availability

The source data for this study are available from the corresponding author upon reasonable request.

## References

[1] Siegel RL , Miller KD , Fuchs HE , Jemal A . Cancer statistics, 2022. CA Cancer J Clin. 2022;72(1):7‐33. 35020204 10.3322/caac.21708

[2] Stravopodis DJ , Papavassiliou KA , Papavassiliou AG . Vistas in non‐small cell lung cancer (NSCLC) treatment: of Kinome and signaling networks. Int J Biol Sci. 2023;19(7):2002‐2005. 37151885 10.7150/ijbs.83574 PMC10158021

[3] Gridelli C , Rossi A , Carbone DP , et al. Non‐small‐cell lung cancer. Nat Rev Dis Primers. 2015;1:15009. 27188576 10.1038/nrdp.2015.9

[4] Herbst RS , Morgensztern D , Boshoff C . The biology and management of non‐small cell lung cancer. Nature. 2018;553(7689):446‐454. 29364287 10.1038/nature25183

[5] Arbour KC , Riely GJ . Systemic therapy for locally advanced and metastatic non–small cell lung cancer: a review. JAMA. 2019;322(8):764‐774. 31454018 10.1001/jama.2019.11058

[6] Rhodin KE , Rucker AJ , Ready NE , D'Amico TA , Antonia SJ . The immunotherapeutic landscape in non‐small cell lung cancer and its surgical horizons. J Thorac Cardiovasc Surg. 2020;159(4):1616‐1623. 31836182 10.1016/j.jtcvs.2019.08.138

[7] Vetting MW , S de Carvalho LP , Yu M , et al. Structure and functions of the GNAT superfamily of acetyltransferases. Arch Biochem Biophys. 2005;433(1):212‐226. 15581578 10.1016/j.abb.2004.09.003

[8] Lv J , Liu H , Wang Q , Tang Z , Hou L , Zhang B . Molecular cloning of a novel human gene encoding histone acetyltransferase‐like protein involved in transcriptional activation of hTERT. Biochem Biophys Res Commun. 2003;311(2):506‐513. 14592445 10.1016/j.bbrc.2003.09.235

[9] Liu X , Tan Y , Zhang C , et al. NAT10 regulates p53 activation through acetylating p53 at K120 and ubiquitinating Mdm2. EMBO Rep. 2016;17(3):349‐366. 26882543 10.15252/embr.201540505 PMC4772976

[10] Shen Q , Zheng X , McNutt MA , et al. NAT10, a nucleolar protein, localizes to the midbody and regulates cytokinesis and acetylation of microtubules. Exp Cell Res. 2009;315(10):1653‐1667. 19303003 10.1016/j.yexcr.2009.03.007

[11] Cai S , Liu X , Zhang C , Xing B , Du X . Autoacetylation of NAT10 is critical for its function in rRNA transcription activation. Biochem Biophys Res Commun. 2017;483(1):624‐629. 27993683 10.1016/j.bbrc.2016.12.092

[12] Liu X , Cai S , Zhang C , et al. Deacetylation of NAT10 by Sirt1 promotes the transition from rRNA biogenesis to autophagy upon energy stress. Nucleic Acids Res. 2018;46(18):9601‐9616. 30165671 10.1093/nar/gky777 PMC6182161

[13] Sleiman S , Dragon F . Recent advances on the structure and function of RNA acetyltransferase Kre33/NAT10. Cells‐Basel. 2019;8(9):1035. 10.3390/cells8091035 PMC6770127 31491951

[14] Zhang H , Hou W , Wang H , et al. GSK‐3β–regulated N‐acetyltransferase 10 is involved in colorectal cancer invasion. Clin Cancer Res. 2014;20(17):4717‐4729. 24982245 10.1158/1078-0432.CCR-13-3477

[15] Ma R , Chen J , Jiang S , Lin S , Zhang X , Liang X . Up regulation of NAT10 promotes metastasis of hepatocellular carcinoma cells through epithelial‐to‐mesenchymal transition. Am J Transl Res. 2016;8(10):4215‐4223. 27830005 PMC5095314

[16] Oh T , Lee Y , Lim B , Lim J . Inhibition of NAT10 suppresses Melanogenesis and melanoma growth by attenuating Microphthalmia‐associated transcription factor (MITF) expression. Int J Mol Sci. 2017;18(9):1924. 28880216 10.3390/ijms18091924 PMC5618573

[17] Xu D , Huang K , Chen Y , Yang F , Xia C , Yang H . Immune response and drug therapy based on ac4C‐modified gene in pancreatic cancer typing. Front Immunol. 2023;14:1133166. 36949954 10.3389/fimmu.2023.1133166 PMC10025374

[18] Pan Z , Bao Y , Hu M , et al. Role of NAT10‐mediated ac4C‐modified HSP90AA1 RNA acetylation in ER stress‐mediated metastasis and lenvatinib resistance in hepatocellular carcinoma. Cell Death Dis. 2023;9(1):56. 10.1038/s41420-023-01355-8 PMC9918514 36765042

[19] Larrieu D , Britton S , Demir M , Rodriguez R , Jackson SP . Chemical inhibition of NAT10 corrects defects of laminopathic cells. Science. 2014;344(6183):527‐532. 24786082 10.1126/science.1252651 PMC4246063

[20] Wu J , Zhu H , Wu J , Chen W , Guan X . Inhibition of N‐acetyltransferase 10 using remodelin attenuates doxorubicin resistance by reversing the epithelial‐mesenchymal transition in breast cancer. Am J Transl Res. 2018;10(1):256‐264. 29423010 PMC5801363

[21] Tsai K , Jaguva Vasudevan AA , Martinez Campos C , Emery A , Swanstrom R , Cullen BR . Acetylation of cytidine residues boosts HIV‐1 gene expression by increasing viral RNA stability. Cell Host Microbe. 2020;28(2):306‐312. 32533923 10.1016/j.chom.2020.05.011 PMC7429276

[22] Dalhat MH , Mohammed M , Ahmad A , Khan MI , Choudhry H . Remodelin, a N‐acetyltransferase 10 (NAT10) inhibitor, alters mitochondrial lipid metabolism in cancer cells. J Cell Biochem. 2021;122(12):1936‐1945. 34605570 10.1002/jcb.30155

[23] Miller M , Hanna N . Advances in systemic therapy for non‐small cell lung cancer. BMJ. 2021;375:n2363. 34753715 10.1136/bmj.n2363

[24] Wang M , Herbst RS , Boshoff C . Toward personalized treatment approaches for non‐small‐cell lung cancer. Nat Med. 2021;27(8):1345‐1356. 34385702 10.1038/s41591-021-01450-2

[25] Reck M , Remon J , Hellmann MD . First‐line immunotherapy for non‐small‐cell lung cancer. J Clin Oncol. 2022;40(6):586‐597. 34985920 10.1200/JCO.21.01497

[26] Hirsch FR , Scagliotti GV , Mulshine JL , et al. Lung cancer: current therapies and new targeted treatments. Lancet. 2017;389(10066):299‐311. 27574741 10.1016/S0140-6736(16)30958-8

[27] Mithoowani H , Febbraro M . Non‐small‐cell lung cancer in 2022: a review for general practitioners in oncology. Curr Oncol. 2022;29(3):1828‐1839. 35323350 10.3390/curroncol29030150 PMC8946954

[28] Ito S , Horikawa S , Suzuki T , et al. Human NAT10 is an ATP‐dependent RNA acetyltransferase responsible for N4‐acetylcytidine formation in 18 S ribosomal RNA (rRNA). J Biol Chem. 2014;289(52):35724‐35730. 25411247 10.1074/jbc.C114.602698 PMC4276842

[29] Sharma S , Langhendries J , Watzinger P , Kötter P , Entian K , Lafontaine DLJ . Yeast Kre33 and human NAT10 are conserved 18S rRNA cytosine acetyltransferases that modify tRNAs assisted by the adaptor Tan1/THUMPD1. Nucleic Acids Res. 2015;43(4):2242‐2258. 25653167 10.1093/nar/gkv075 PMC4344512

[30] Arango D , Sturgill D , Alhusaini N , et al. Acetylation of cytidine in mRNA promotes translation efficiency. Cell. 2018;175(7):1872‐1886. 30449621 10.1016/j.cell.2018.10.030 PMC6295233

[31] Jin G , Xu M , Zou M , Duan S . The processing, gene regulation, biological functions, and clinical relevance of N4‐Acetylcytidine on RNA: a systematic review. Mol Ther Nucleic Acids. 2020;20:13‐24. 32171170 10.1016/j.omtn.2020.01.037 PMC7068197

[32] Feng Z , Li K , Qin K , et al. The LINC00623/NAT10 signaling axis promotes pancreatic cancer progression by remodeling ac4C modification of mRNA. J Hematol Oncol. 2022;15(1):112. 35978332 10.1186/s13045-022-01338-9 PMC9387035

[33] Zhang Y , Jing Y , Wang Y , et al. NAT10 promotes gastric cancer metastasis via N4‐acetylated COL5A1. Signal Transduct Target Ther. 2021;6(1):173. 33941767 10.1038/s41392-021-00489-4 PMC8093205

[34] Liao L , He Y , Li SJ , et al. Lysine 2‐hydroxyisobutyrylation of NAT10 promotes cancer metastasis in an ac4C‐dependent manner. Cell Res. 2023;33(5):355‐371. 36882514 10.1038/s41422-023-00793-4 PMC10156899

[35] Larrieu D , Viré E , Robson S , Breusegem SY , Kouzarides T , Jackson SP . Inhibition of the acetyltransferase NAT10 normalizes progeric and aging cells by rebalancing the Transportin‐1 nuclear import pathway. Sci Signal. 2018;11(537):r5401. 10.1126/scisignal.aar5401 PMC6331045 29970603

