# Supplementary material for: Remodelin delays non‐small cell lung cancer progression by inhibiting NAT10 via the EMT pathway
Source: Cancer Med. 2024 Jun 3;13(11):e7283. doi: 10.1002/cam4.7283 (PMC11145023; doi:10.1002/cam4.7283)
Supplement: Supplementary file 1 — Table S1: [file CAM4-13-e7283-s001.docx]

**Supporting Information**

**Table S1: Clinicopathological characteristics of patient samples and expression of NAT10 in NSCLC**

| **Characteristics** | | **Number of cases (%)** |
| --- | --- | --- |
| **Age** | ≥60 | 53(54.08%) |
|  | ＜60 | 45(45.92%) |
| **Gender** | Male | 55(56.12%) |
|  | Female | 43(43.88%) |
| **clinical stage** | I | 34(34.69%) |
|  | II | 28(28.57%) |
|  | III | 36(36.74%) |
| **T classifcation** | 1 | 20(20.41%) |
|  | 2 | 52(53.06%) |
|  | 3 | 21(21.43%) |
|  | 4 | 5(5.10%) |
| **N classifcation** | 0 | 45(45.92%) |
|  | 1 | 26(26.53%) |
|  | 2 | 21(21.43%) |
|  | 3 | 6(6.12%) |
| **Vital states**  **(at follow-up)** | Alive or lost | 23(23.47%) |
|  | Dead | 75(76.53%) |
| **Expression of NAT10** | Low expression | 44(44.90%) |
|  | High expression | 54(55.1%) |
