# Supplementary material for: Remodelin delays non‐small cell lung cancer progression by inhibiting NAT10 via the EMT pathway
Source: Cancer Med. 2024 Jun 3;13(11):e7283. doi: 10.1002/cam4.7283 (PMC11145023; doi:10.1002/cam4.7283)
Supplement: Supplementary file 2 — Table S2: [file CAM4-13-e7283-s003.docx]

**Supporting Information**

**Table S2: Correlation between NAT10 expression and clinicopathologic characteristics of NSCLC patients**

| **Characteristics** | | **NAT10 expression** | | ***P* value** |
| --- | --- | --- | --- | --- |
|  |  | Low expression | High expression |  |
| **Age** | ≥60 | 21（39.62%） | 32（60.38%） | 0.25 |
|  | ＜60 | 23（51.11%） | 22（48.89%） |  |
| **Gender** | Male | 23（41.82%） | 32（58.18%） | 0.48 |
|  | Female | 21（48.84%） | 22（51.16%） |  |
| **clinical stage** | I+II | 44（71.97%） | 18（29.03%） | <0.01 |
|  | III | 0（0.00%） | 36（100.00%） |  |
| **T classifcation** | T1+T2 | 39(54.17%) | 33(45.83%) | <0.01 |
|  | T3+T4 | 5(19.23%) | 21(80.87%) |  |
| **Lymph node metastasis** | No | 44(97.78%) | 1(2.22%) | <0.01 |
|  | Yes | 0(0.00%) | 53(100.00%) |  |
| **Vital states**  **(at follow-up)** | Alive or lost | 15(65.22%) | 8(34.78%) | 0.03 |
|  | Dead | 29(38.67%) | 46(61.33%) |  |
