# Supplementary material for: Remodelin delays non‐small cell lung cancer progression by inhibiting NAT10 via the EMT pathway
Source: Cancer Med. 2024 Jun 3;13(11):e7283. doi: 10.1002/cam4.7283 (PMC11145023; doi:10.1002/cam4.7283)
Supplement: Supplementary file 3 — Table S3: [file CAM4-13-e7283-s002.docx]

**Supporting Information**

**Table S3: The NAT10 expression in tumor and normal lung tissue**

|  | NAT10 high expression | NAT10 low expression |
| --- | --- | --- |
| Tumor | 54 | 44 |
| Normal | 1 | 81 |
